# Characterizing the skeletal muscle immune microenvironment for sarcopenia: insights from transcriptome analysis and histological validation

**DOI:** 10.3389/fimmu.2024.1414387

**Published:** 2024-07-04

**Authors:** Linhui Shen, Yuan Zong, Jiawen Zhao, Yi Yang, Lei Li, Ning Li, Yiming Gao, Xianfei Xie, Qiyuan Bao, Liting Jiang, Weiguo Hu

**Affiliations:** ^1^ Department of Geriatrics, Ruijin hospital, Shanghai Jiao Tong University School of Medicine, Shanghai, China; ^2^ Department of Stomatology, Ruijin Hospital, Shanghai Jiao Tong University School of Medicine, College of Stomatology, Shanghai Jiao Tong University, Shanghai, China; ^3^ Department of Pathology, Ruijin Hospital, Shanghai Jiao Tong University School of Medicine, Shanghai, China; ^4^ Hainan Branch, Ruijin Hospital, Shanghai Jiao Tong University School of Medicine, Qionghai, China; ^5^ Department of Orthopaedics, Shanghai Ruijin Hospital, Shanghai Jiao Tong University School of Medicine, Shanghai, China; ^6^ Department of Orthopaedics, Shanghai Key Laboratory for Prevention and Treatment of Bone and Joint Diseases, Shanghai Institute of Traumatology and Orthopaedics, Ruijin Hospital, Shanghai Jiao Tong University School of Medicine, Shanghai, China; ^7^ Medical Center on Aging of Ruijin Hospital, Shanghai Jiao Tong University School of Medicine, Shanghai, China

**Keywords:** sarcopenia, skeletal muscle, immune microenvironment, single-cell RNA sequencing, transcriptomics, cell communication, inflammation

## Abstract

**Background:**

Sarcopenia is a condition characterized by the age-related loss of skeletal muscle mass and function. The pathogenesis of the disease is influenced by chronic low-grade inflammation. However, the specific changes in the immune landscape changes of sarcopenic muscle are not yet fully understood.

**Methods:**

To gain insights into the immune cell composition and interactions, we combined single-nucleus RNA sequencing data, bulk RNA sequencing dataset, and comprehensive bioinformatic analyses on the skeletal muscle samples from young, aged, and sarcopenic individuals. Histological staining was then performed on skeletal muscles to validate the distribution of immune cells in clinical samples.

**Results:**

We analyzed the transcriptomes of 101,862 single nuclei, revealing a total of 10 major cell types and 6 subclusters of immune cell types within the human skeletal muscle tissues. Notable variations were identified in the immune microenvironment between young and aged skeletal muscle. Among the immune cells from skeletal muscle microenvironment, macrophages constituted the largest fraction. A specific marker gene LYVE1 for skeletal muscle resident macrophages was further identified. Cellular subclasses included four distinct groups of resident macrophages, which play different roles in physiological or non-physiological conditions. Utilizing bulk RNA sequencing data, we observed a significant enrichment of macrophage-rich inflammation in sarcopenia.

**Conclusions:**

Our findings demonstrate age-related changes in the composition and cross-talk of immune cells in human skeletal muscle microenvironment, which contribute to chronic inflammation in aged or sarcopenia muscle. Furthermore, macrophages emerge as a potential therapeutic target, thus advancing our understanding of the pathogenesis of sarcopenia.

## 1 Introduction

Sarcopenia is a systemic skeletal muscle disease that occurs with aging, characterized by the progressive loss of muscle mass and function (1). It is commonly associated with weakness, immobility, and premature death (2). Similar to other human tissues, skeletal muscle degeneration also happens with age (1). The incidence of sarcopenia increases as individuals get older, with an average onset age of over 70 years old (3–5). This has made sarcopenia a significant public health concern in aging societies (6), as it is linked to numerous adverse outcomes such as falls, fractures, and mortality in the elderly. Histopathologically, sarcopenia is characterized by reductions in the number and size of muscle fibers, especially Type-II muscle fibers, as well as fatty infiltration in skeletal muscle (1, 7). Muscle anabolic resistance, mitochondrial dysfunction, inflammation, and degenerative changes in the nervous system are found to be key factors in the development of sarcopenia (7). However, the exact mechanism underlying the development of sarcopenia is not yet fully understood.

In recent years, there has been a growing interest in studying the relationship between sarcopenia, immunity, and inflammation. As individuals age, they often experience a chronic low-grade pro-inflammatory state characterized by an elevation in pro-inflammatory cytokines and a decline in immune cell function (8). Frail elderly individuals exhibit elevated levels of tumor necrosis factor-α (TNF-α) in comparison to healthy young adults, and interleukin-6 (IL-6) is significantly associated with sarcopenia (9, 10). The elevated expression of these cytokines can contribute to muscle atrophy (11, 12). Additionally, B cells may have a significant impact on the regulation of sarcopenia through transcriptional mechanisms (13). Moreover, Research has shown a correlation between sarcopenia and autoimmune diseases. For instance, rheumatoid arthritis (RA) patients are at an increased risk for sarcopenia (14). Other autoimmune diseases, such as multiple sclerosis, celiac disease, type 1 diabetes, psoriasis, and ulcerative colitis have also been associated with sarcopenia (15). Therefore, further investigation into the interplay among sarcopenia, immunity, and inflammation is warranted.

Chronic inflammation in sarcopenia is a complex process involving various specialized immune cell types. The interactions between immune cells and native cells in skeletal muscle tissue are crucial for both the progression of inflammation and the repair of damage. In patients with sarcopenia, T helper 17 (Th17) cells are down-regulated, while NK cells are up-regulated. Furthermore, certain key genes regulate the progression of sarcopenia by influencing the immune microenvironment (16). When muscle is damaged, resident neutrophils and macrophages become activated, and the cytokines and growth factors released by activated T cells play a vital role in the proliferation and migration of muscle satellite cells (17). However, the number of these cells decreases in aged people, potentially leading to inadequate muscle repair. Despite several studies focusing on specific cell populations in sarcopenia, comprehensive analyses of skeletal muscle immune microenvironment are still lacking.

Single-cell RNA sequencing (scRNA-seq) is a powerful technology that enables precise measurement of gene expression at a cellular level. It provides valuable insights into the status of different cells in the body’s microenvironment, something that traditional transcriptomic technologies are unable to achieve. Due to challenges with skeletal muscle dissociation and cellular filtration, there are limited studies on the single-cell atlas of the skeletal muscle system to date. While some related analyses have been published recently (18–21), they still have a narrow focus. Single-nucleus RNA sequencing (snRNA-seq) has been utilized to capture transcriptomic signatures from mature myofiber nuclei in myofibers, offering comparable gene detection to scRNA-seq and reducing dissociation bias (22). The objective of this study was to investigate the immune microenvironment of skeletal muscle in the young and aged groups using snRNA-seq data and bulk RNA seq sequencing (RNA-seq) dataset. We also examined the gene expression patterns associated with sarcopenia and highlighted the significant role of immune cells in its development. The identification of key genes and cell types may provide a potential therapeutic target for intervening in the progression of sarcopenia.

## 2 Method

### 2.1 Tissue samples collection and cohort description

Patients who underwent surgery due to trauma between June 2023 and January 2024 at the Department of Orthopaedics, Shanghai Ruijin Hospital, were included in the study. Adult human skeletal muscle tissue samples for immunostaining were obtained from 18 patients. These patients were categorized into three groups: young patients (aged 35 years or younger), aged patients without sarcopenia (aged 60 years or older), and aged patients with sarcopenia (aged 60 years or older). Sarcopenia was diagnosed according to the Asian Working Group for Sarcopenia (AWGS) 2019 Criteria (6). Demographic information, including age, gender, medical history, and pathological diagnosis were collected for each patient. All participants have provided the informed consents and the study was approved by the Ethics Committee of Ruijin Hospital, Shanghai Jiao Tong University School of Medicine (IRB number KQ20230608v1.0/2023–06-08).

### 2.2 Data preparation

Single-nucleus RNA transcriptome, bulk RNA transcriptome data and corresponding clinical information were obtained from the Gene Expression Omnibus (GEO) database (https://www.ncbi.nlm.nih.gov/geo/). The dataset GSE167186 contained 72 bulk RNA-seq samples from young, aged and sarcopenic subjects, as well as 17 snRNA-seq samples from young and aged subjects, with a total of 143,051 nuclei included. The study by Perez K et al. detailed the sequencing procedures of snRNA-seq and bulk RNA-seq data used for the muscle biopsy samples (23). LIMMA package was used to normalize the RNA-seq data (24).

### 2.3 snRNA-seq data analysis

The quality control and downstream analysis of snRNA-seq data were performed using the Seurat R package (http://satijalab.org/seurat/). Low-quality nuclei were filtered using the following cutoffs: nFeature_RNA > 500 & nFeature_RNA < 4000 & percent.mt < 18 & nCount_RNA > 500 & nCount_RNA < 10000. This filtering process resulted in 101,862 cells remaining. Thet-SNE algorithm was utilized for cell cluster generation (25). Cell types within the clusters were manually annotated based on cell markers from original publications and well-known markers using SingleR (26). Bubble charts and violin charts were employed to visualize the expression of marker genes in each cluster.

We screened pathways related to functions, metabolism, and immunity processes in skeletal muscle using the kyoto encyclopedia of genes and genomes (KEGG) dataset (https://www.kegg.jp/kegg/) and the Gene Ontology (GO) dataset (https://biit.cs.ut.ee/gprofiler/). Gene set variation analysis (GSVA) was performed using the GSVA R package to characterize the differentially expressed pathways of each cluster (27). The average expression values of genes for each cell type were used as input data. Cell-cell communications (CCCs) among cell clusters in the skeletal muscle microenvironment, especially among immune cell clusters were identified and visualized using the CellChat package.

### 2.4 Bulk RNA-seq data analysis

The Gene Set Enrichment Analysis (GSEA) (https://www.gsea-msigdb.org/gsea/) and pathway signal score calculation was conducted using KEGG, Hallmark, and WIKI-pathway gene sets. Differentially expressed pathways with a normalized (NOM) P-value of <0.05 were kept for further functional analysis. To create heatmaps based on gene expression in young and aged skeletal muscle tissues, we utilized the ggplot2 R package (http://ggplot2.tidyverse.org/) with fragments per kilobase of transcript per million sequenced reads (FPKM) data. The single-sample gene set enrichment analysis (ssGSEA) algorithm was then employed to evaluate the enrichment score of each pathway (28). We used the pheatmap R package to generate the heatmap for correlation coefficients of mitochondria-related and muscle-function related pathways and muscle microenvironment genes. For the correlation analysis, we used the Spearman correlation test.

The ESTIMATE package was used to calculate ESTIMATE, immune, and stromal scores for the RNA-seq data of young, aged, and sarcopenia samples (29), and the enrichment scores calculated by the ssGSEA analysis were used to evaluate immune-related gene activity and the relative abundance of infiltrating immune cells. We then performed deconvolution on our RNA-seq dataset using the CIBERSORT algorithm (http://cibersort.stanford.edu) to estimate the relative fraction of immune cells. The correlation analysis among different immune cell types was conducted using the ggstatsplot package, and the results were visualized using the ggplot2 package.

To identify groups of highly correlated genes, we used the unsupervised method called weighted gene correlation network analysis (WGCNA) (30). The eigengenes from each module were used to assess the relationship between the modules and immune cell types, as well as clinical information. For functional annotation analyses, we utilized the online tool Metascape (http://metascape.org) (31). The network of enriched term subsets was visualized using Cytoscape v3.10.1 (https://cytoscape.org/). GeneMINIA (http://www.genemania.org) was used to predict the functional association network of hub genes (32).

### 2.5 Histological analysis

Fresh muscle samples were fixed in 10% neutral buffered formalin overnight at room temperature. The 4μm thick paraffin-embedded tissue sections were air-dried, followed by additional drying at 75°C for 2 hours, and then dewaxed. Hematoxylin and eosin (H&E) staining was carried out using a CoverStainer (Dako, Germany) following standard procedures. Immunohistochemical (IHC) procedures were performed on an automated Leica Bond RX staining platform (Leica Biosystems, Welzlar, Germany) following the selected protocol for the BOND Polymer Refine Detection kit (DS9800, Leica Biosystems). For a list of primary antibodies, please refer to **Supplementary Table S1**. For Periodic acid-Schiff (PAS) staining, the sections were incubated in Periodate solution for 5 minutes, and then stained with Schiff reagent using a PAS Staining Kit following a protocol suggested by the manufacturer. Images were captured using a Nikon Eclipse Ni-U microscope equipped with a Nikon DS-Ri digital camera. Immunofluorescence (IF) staining was performed on skeletal muscle tissue samples as previously described (33). Representative fluorescence images were captured on a Leica TCS SP8 MP confocal microscope (Leica Microsystems, Wetzlar Germany).

### 2.6 Statistical analysis

All data were expressed as the mean ± standard deviation (SD). R (version 4.2.3) and GraphPad Prism (version 8) were used for statistical analysis. The t-test or one-way ANOVA test was utilized for group comparisons, while the Spearman correlation test was employed to assess the correlation between variables. P-values less than 0.05 were considered statistically significant.

## 3 Results

### 3.1 Cell type composition of skeletal muscle tissue from young and aged people

**Figure 1:**
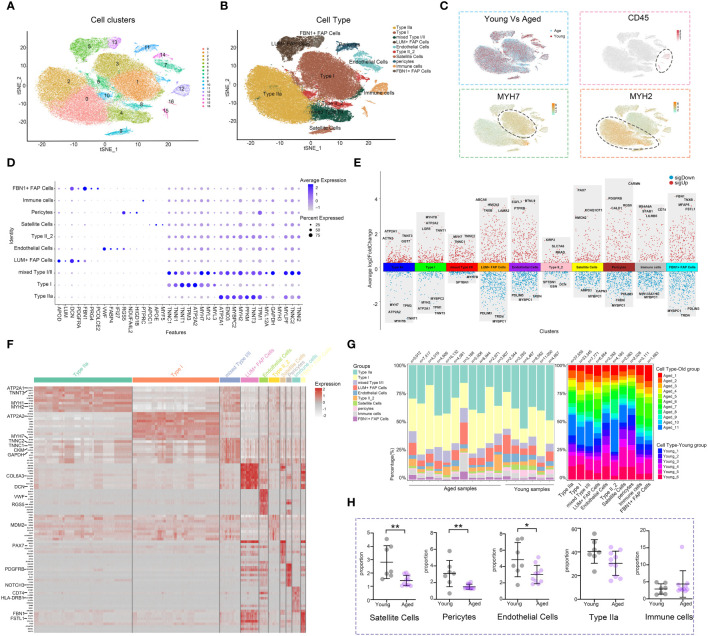
Analysis of the cell composition of young and aged skeletal muscle based on snRNA-seq data. **(A)** t-distributed stochastic neighbor embedding (t-SNE) plot of joint analysis of cell composition in young and aged skeletal muscle tissue. 16 unsupervised clusters were generated from 101,862 cell nuclei after quality control (distinguished by different colors and numbers). **(B)** Following dimensionality reduction, 10 clusters were manually annotated based on marker gene expression and visualized with t-SNE. **(C)** Feature plots (t-SNE) showing expressions of major genes that identify immune cell subpopulations. **(D)** Dot plots representing the percentage and average expression of selected marker genes for each cluster. **(E)** Differentially expressed genes (DEGs) in each cell type in aged versus young skeletal muscle. **(F)** Heatmap depicting the expression of the top 10 upregulated genes identified in each cell cluster. Each row represents a single cell, and each column represents a single gene. **(G)** Bar plot depicting the proportion of each cell subset in each sample and the proportion of each sample in each cell subset. **(H)** Cell proportion analysis of satellite cells, pericytes, endothelial cells, Type IIa and immune cells between the young and aged (*p<0.05; **p< 0.01).

The snRNA-seq dataset from GSE167186 included skeletal muscle tissue samples from 6 young and 11 aged individuals. Through snRNA-seq analysis, we were able to identify the age‐related changes in the cellular composition of skeletal muscle tissue. After performing quality control, we retained 101,862 nuclei for further analysis. Unsupervised classification partitioned the 101,862 nuclei into 17 clusters (**Figure 1A**). Based on the canonical gene markers, we manually assigned these clusters to 9 cell types: Type IIa, Type I, LUM+ Fibro-Adipogenic Progenitor (FAP) Cells, Endothelial Cells, Type II_2, ls, Pericytes, Immune cells and FBN1+ FAP Cells (**Figures 1B, D**, **Supplementary Figure S3A**). The study identified a cluster that exhibited characteristics of both Type I and Type II, referred to as mixed Type I/II. Differences in the numbers and distribution of different cell clusters in aged and young muscle, as well as the expression level and distribution of gene markers of immune cells, type I and type II muscle fiber were illustrated (**Figure 1C**). The volcano diagram displayed the top differentially expressed genes (DEGs) in each cell type, revealing extensive transcriptomic alterations in cell function and metabolism in the aging muscle (**Figures 1E**, **Supplementary Figure S3B**; **Additional File 1**: **Supplementary Table S2**). The heatmap presented the top-enriched genes in each cluster (**Figures 1F**, **Supplementary Figure S3C**). Type I and type II muscle cells were observed to be the main components in skeletal muscle tissue, although their composition varied between young and aged individuals (**Figure 1G**). Notably, the proportion of type IIa muscle cells showed a general downward trend in the aged group, and there was a significant decrease in endothelial cells, satellite cells, and pericytes compared to the young group. While not statistically significant, there was an observed trend of increasing immune cell numbers in the aged group. (**Figure 1H**). The decreased number and fibrotic transformation of satellite cells in aging muscle might contribute to a decreased muscle regeneration capacity (34). Previous studies have demonstrated a decrease in angiogenesis and blood flow in aged muscle tissue, which is associated with the development of sarcopenia (35). Further investigations are needed to understand the changes in immune cell subsets and metabolism in the muscle aging microenvironment, considering the role of chronic inflammation in sarcopenia development.

### 3.2 Immune cell composition in skeletal muscle tissue

**Figure 2:**
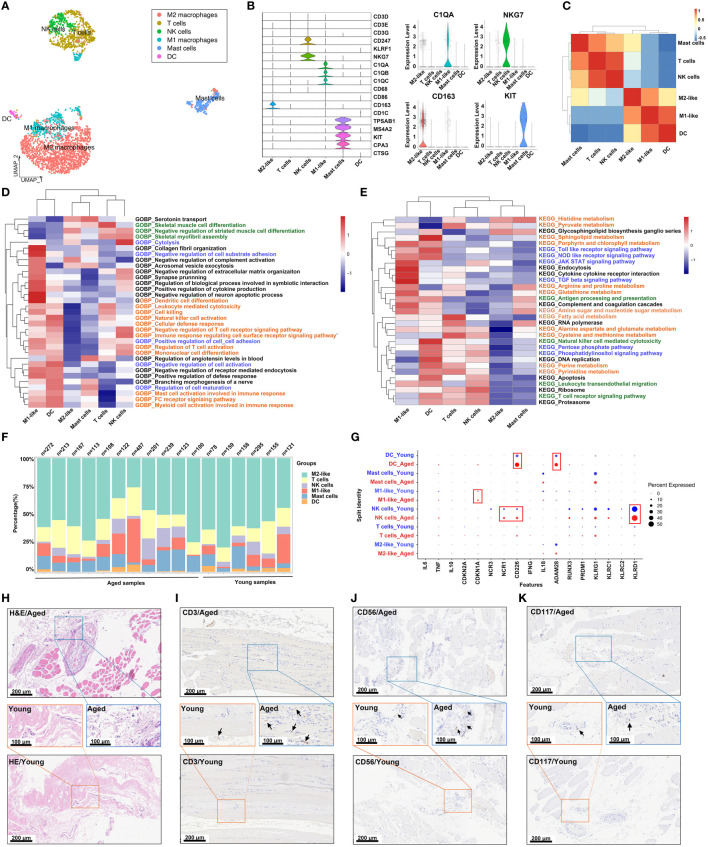
Analysis of the immune cell subpopulations of young and aged skeletal muscle based on snRNA-seq data. **(A)** Uniform manifold approximation and projection (UMAP) plot of 6 immune cell types for joint analysis in young and aged skeletal muscle tissue. **(B)** Violin plots of expression values for cell type‐specific marker genes. **(C)** Heatmap of Spearman’s correlations among immune cell subtypes. Correlation coefficient values are color-coded from blue (-0.5) to red (1.0) using the color bar on the right. **(D, E)** Comparison of pathway enrichment across immune subpopulations. Heatmap of enrichment scores of selected Gene Ontology (GO) **(D)** and kyoto encyclopedia of genes and genomes (KEGG) **(E)** pathways in immune cell clusters following gene set variation analysis (GSVA). **(F)** Bar plot depicting the proportion of each immune cell subset in each sample. **(G)** Dot plots demonstrating the expression differences of inflammation-associated genes in each cluster in the young group versus aged individuals. **(H)** Representative images of hematoxylin and eosin (H&E) staining in young and aged skeletal muscle specimens. **(I–K)** Representative images of immunohistochemical (IHC) staining for CD3 **(I)**, CD56 **(J)** and CD117 **(K)** (scale bar = 100 μm).

In order to explore the role of immune cells in the aging muscle microenvironment, we further grouped immune cells into 6 clusters: M2 macrophage, T cell, NK cell, M1 macrophage, mast cell and dendritic cell (DC) (**Figure 2A**). The expression profiles of marker genes for different cell populations were depicted in a violin plot (**Figure 2B**). Subsequently, we evaluated the interactions among immune cells. In the skeletal muscle microenvironment, significant positive correlations were observed between DCs and M1 macrophages, and between NK cells and T cells, while a negative correlation was observed between DCs and mast cells (**Figure 2C**), which aligned with the proximity depicted in the UMAP. Furthermore, various functional and metabolic pathways were found to be enriched in different subtypes of immune cells. GO and KEGG pathway analysis revealed enrichment for terms such as collagen fibril organization, DC differentiation, JAK-STAT signaling pathway, endocytosis and TGF-β signaling pathway in M1-like macrophages. Similarly, T cells showed enrichment in terms related to skeletal muscle cell differentiation, fatty acid metabolism, cysteine and methionine metabolism. The NK cell population exhibited high enrichment in cytolysis and ribosome, while DCs showed enrichment in sphingolipid metabolism and natural killer cell mediated cytotoxicity (**Figures 2D, E**; **Additional File 2**: **Supplementary Table S3**). The skeletal muscle immune microenvironment was a complex milieu consisting of several innate and adaptive immune cells, with innate immune cells being the majority. Among the innate immune cells, M1 and M2 macrophages were the most abundant (**Figure 2F**). Furthermore, the expression levels of some cell-specific marker genes involved in the immune response and aging process showed differences between the young and aged groups (**Figure 2G**). For instance, the aged group exhibited increased CD226+ and ADAM58+ cell presence in DCs, CDKN1A+ cell presence in M1 macrophages, and NCR1+, CD226+ and KLRD1+ cell presence in NK cells. Histological staining revealed an augmentation in immune cells within the aged samples (**Figures 2H–K**). H&E staining indicated that the interstitial tissue of aged muscles exhibited a loose and fragmented structure, accompanied by a higher distribution of lymphocytes (**Figure 2H**), while IHC staining confirmed the higher presence of T (CD3+), NK (CD56+), and hematopoietic stem and progenitor (CD117+) cells in the aged skeletal muscle samples (**Figures 2I–K**, **Supplementary Figures S2A–C**). Given the critical position of the skeletal muscle immune microenvironment, we next sought to investigate the differences in macrophage expression and functions between the young and aged groups.

### 3.3 Macrophage subpopulations in skeletal muscle tissue

**Figure 3:**
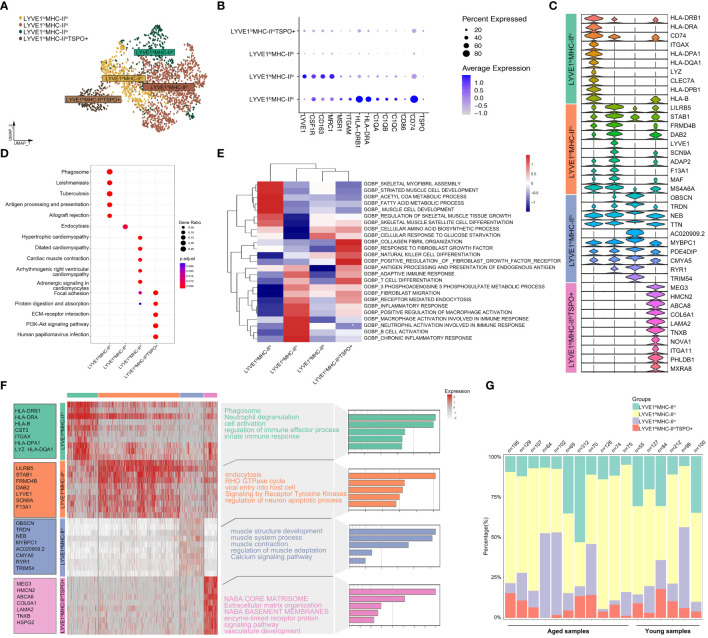
Deep profiling of the macrophage subpopulations in skeletal muscle microenvironment based on snRNA-seq data. **(A)** UMAP plot of 4 macrophage subtypes in skeletal muscle tissue. **(B)** Dot plots representing the expression levels of selected marker genes for each macrophage cluster. **(C)** Violin plots of expression values for selected marker genes for each cluster. **(D)** KEGG enrichment analysis revealed biological processes associated with each subset. **(E)** Heatmap of enrichment scores of selected GO pathways in macrophage clusters following GSVA. **(F)** Heatmap depicting the expression of the top upregulated genes identified in each macrophage cluster. Each row represents a single cell, and each column represents a single gene. The left side displays the specific gene names, and the right side lists the pathways associated with these genes (inferred utilizing Metascape). **(G)** Bar plot depicting the proportion of each macrophage subset in each sample.

Macrophages were classified into four subgroups based on the expression levels of the selected marker genes LYVE1, HLA-DRB1, HLA-DRA and TSPO. These subgroups were LYVE1^lo^MHC-II^hi^, LYVE1^hi^MHC-II^lo^, LYVE1^lo^MHC-II^lo^ and LYVE1l^lo^MHC-II^lo^TSPO^+^ (36, 37) (**Figures 3A, B**). A violin plot was used to visualize the gene signatures expressed by these subpopulations (**Figure 3C**). Subsequently, GO and KEGG analyses were conducted to investigate the functions of the four subpopulations. The results revealed that pathways related to phagosome, antigen processing and presentation, and immune cell activation were highly enriched in the LYVE1^lo^MHC-II^hi^ subpopulation. This suggests that LYVE1^lo^MHC-II^hi^ macrophages play an active role in the immune response. In contrast, LYVE1^hi^MHC-II^lo^ showed significant enrichment in pathways related to endocytosis, amino acid biosynthetic process and cellular response to glucose starvation. LYVE1^lo^MHC-II^lo^ exhibited upregulation in pathways associated with cardiac muscle contraction, skeletal myofibril assembly, acetyl CoA metabolic process and fatty acid metabolic process pathways. On the other hand, LYVE1l^lo^MHC-II^lo^TSPO^+^ displayed upregulation in pathways related to focal adhesion, protein digestion and absorption, ECM-receptor interaction, PI3K-Akt signaling pathway, collagen fibril organization, and response to fibroblast growth factor, indicating a close connection with muscle fibrosis (**Figures 3D, E**; **Additional File 3**: **Supplementary Table S4**). The significantly altered genes within each cluster were included as features for further functional enrichment analysis. The results revealed that innate immune response was associated with LYVE1^lo^MHC-II^hi^, while muscle structure development was associated with LYVE1^lo^MHC-II^lo^, and extracellular matrix organization was associated with LYVE1l^lo^MHC-II^lo^TSPO^+^ (**Figure 3F**). Furthermore, LYVE1^hi^MHC-II^lo^ macrophages were found to be the predominant component of macrophages in the skeletal muscle microenvironment (**Figure 3G**). Meanwhile, the LYVE1^hi^MHC-II^lo^ subpopulation exhibits an increasing trend in the macrophage composition of aging muscles (**Supplementary Figure S2E**).

### 3.4 Cell-cell communication in muscle immune microenvironment

**Figure 4:**
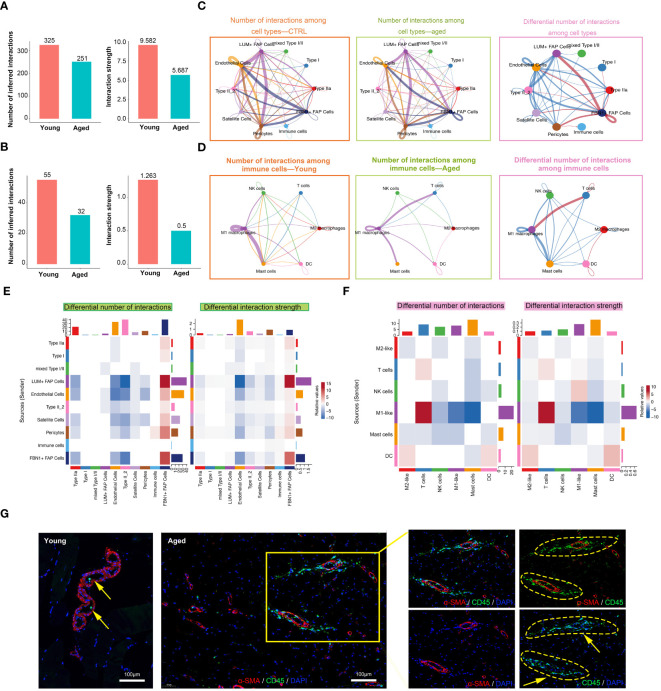
Cell-cell communication in muscle immune microenvironment. **(A, B)** Intercellular ligand–receptor prediction among cell types and immune cells revealed by CellChat. Bar plot showing the number and strength of intercellular interactions among cell types **(A)** and immune cells **(B)**. **(C, D)** Circle plots demonstrating the overview of cell–cell interactions among cell types **(C)** and immune cells **(D)**. Arrow and edge color indicate direction and sender. Edge thickness reflects the number and the strength of interaction between populations. Differential number of interactions in the cell–cell communication network with red or blue colored edges representing increased or decreased signaling in the aged group compared to the young group. Line thickness and darkness indicate the relative enrichment value. **(E, F)** Heatmaps of differential number and strength of intercellular interactions among cell types **(E)** and immune cells **(F)**. **(G)** Representative images of co-IF analysis of immune cells (CD45, green), blood vessels (α-SMA, red) and nuclei (DAPI, blue) of skeletal muscle tissues from the young and aged (scale bar = 100 μm).

The numbers and strength of interactions between major cell types and immune cells were found to be lower in the aged group, indicating decreased cellular communication in the microenvironment of aged skeletal muscle (**Figures 4A, B**). Specifically, the aged group showed a decrease in the interactions between endothelial cells and Type II muscle fibers, while there was a significant increase in the signaling patterns received by FBN1+ FAP Cells. Additionally, a unique signaling pathway was discovered, where pericytes sent signals to and received signals from immune cells (**Figures 4C, E**). When focusing on immune cells, overall interactions among immune cell types decreased in the aged group, but new communications from M1 macrophages to T cells and DC cells, and from T cells to M2 macrophages emerged (**Figures 4D, F**). Next, we performed IF staining to show the inflammatory infiltrates in young and aged muscle. The walls of small blood vessels were labeled with α-SMA. Our findings revealed a significant increase in the number of CD45-labeled macrophages in the aged group compared to the young group (**Figure 4G**; **Supplementary Figure S2D**). Furthermore, we observed a close association between the distribution of these macrophages and small blood vessels. In general, the interaction between cell populations in aged muscle exhibited a decrease compared to the young group, suggesting that the aged microenvironment disrupts normal intercellular communication.

### 3.5 The enrichment of inflammatory signaling pathways in the sarcopenic tissue microenvironment

**Figure 5:**
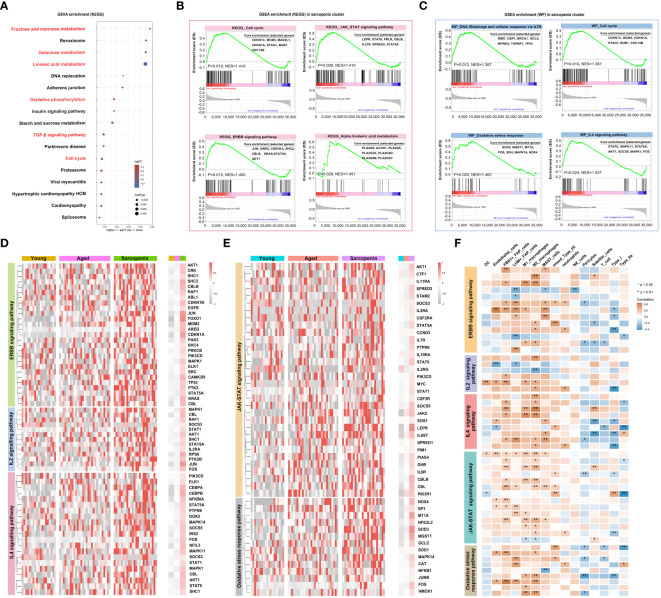
Inflammation is a vital pathophysiological mechanism for sarcopenia. **(A)** Visualization of GSVA for pathways enriched in samples from patients with sarcopenia as compared to samples without sarcopenia using a bubble plot with the color coding indicating the −log10 (p adjusted value), and the size representing the number of genes detected in each pathway. **(B, C)** Hallmark and WIKI-pathway (WP) analysis on sarcopenia patient cohort. NES, normalized enrichment score. **(D, E)** Heatmap demonstrating the expression of genes associated with inflammation-related pathways and pathways related to muscle function across the young, aged and sarcopenia groups based on RNA-seq data. **(F)** Heatmap demonstrating the correlation of inflammation-related pathways and pathways related to muscle function with the skeletal muscle microenvironment in the young, aged and sarcopenia groups based on RNA-seq data. Correlation coefficient values are color-coded from blue (-0.4) to orange (0.4) using the color bar on the right.

In this study, we utilized bulk RNA-seq analysis to investigate the functional and metabolic changes between individuals with sarcopenia and those without. Several pathways were significantly enriched in patients with sarcopenia, such as linoleic acid metabolism, TGF-β signaling and cell cycle pathways, indicating metabolic and immune changes. On the other hand, compared to the young group, several pathways were downregulated in the sarcopenia group, including fructose and mannose metabolism, galactose metabolism and oxidative phosphorylation pathways (**Figure 5A**). To further understand the disease process of sarcopenia, we performed GSEA on RNA-seq data and identified additional biological pathways that could contribute to sarcopenia. The GSEA results confirmed the enrichment of KEGG genes in cell cycle, JAK-STAT signaling pathway, ERBB signaling pathway and alpha-linolenic acid metabolism in the sarcopenia group (**Figure 5B**). We also observed enrichment of WIKI-pathway genes in cell cycle, as well as other pathways such as DNA IRdamage and cellular response via ATR, oxidative stress response and IL-4 signaling pathway in the sarcopenia group (**Figure 5C**). Moreover, we investigated the gene expression of inflammation-related pathways and pathways related to muscle function. The results revealed significantly higher expression of genes associated with ERBB, IL-2, IL-4, oxidative stress response and JAK-STAT signaling pathway in the sarcopenia group (**Figures 5D, E**). Interestingly, these genes were generally down-regulated in the aged group and up-regulated in the sarcopenic group compared to young individuals. Furthermore, the correlation analysis revealed that most genes had a significant negative correlation with FAP cells and macrophages, while satellite cells, Type IIa, and Type I showed an overall opposite trend (**Figure 5F**). In summary, the findings suggested that sarcopenia was strongly associated with increased inflammation and impaired muscle function, which was linked to FAP cells and macrophages.

### 3.6 Immune cell infiltration features of sarcopenia

**Figure 6:**
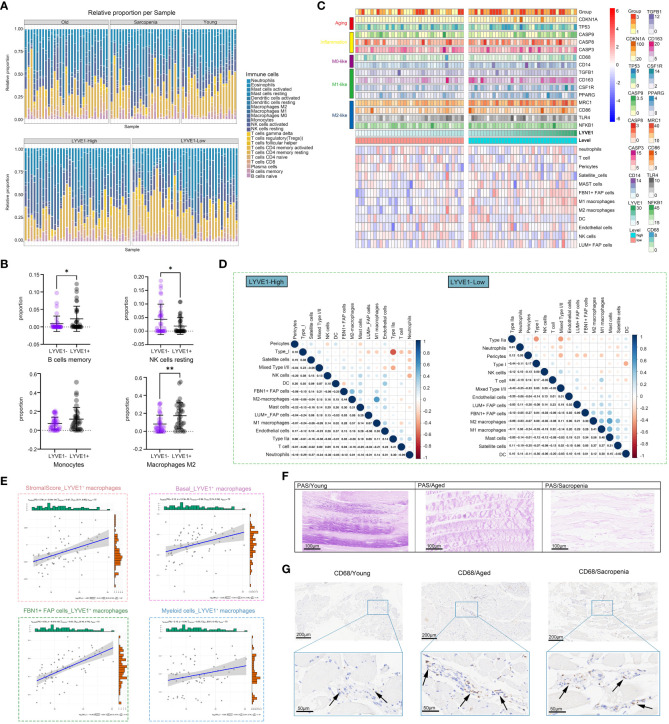
LYVE1 expression is strongly associated with the skeletal muscle immune microenvironment. **(A)** The proportion of tissue-infiltrating innate and adaptive immune cells assessed using the CIBERSORT algorithm is demonstrated in a stacked bar plot. The groups are categorized as young, aged, and sarcopenia patients, and further grouped based on high and low LYVE1 expression. **(B)** Cell proportion analysis of B cells memory, NK cells resting, monocytes and macrophages M2 between the LYVE1-high (LYVE1+) and LYVE1-low (LYVE1-) group (*p<0.05; **p< 0.01). **(C)** Heatmap demonstrating the distribution of immune cell infiltration in the LYVE1-high and LYVE1-low groups generated based on the results, obtained from the ssGSEA and ESTIMATE algorithms and unsupervised hierarchical clustering. Each color represents a gene as indicated on the right, with the colors ranging from light to dark indicating low to high levels of relative gene expression. **(D)** Heatmap showing the relationship between the infiltration levels of various innate and adaptive immune cells in the LYVE1-high and LYVE1-low groups. The Pearson correlation coefficients are provided, with blue indicating a positive correlation and orange indicating a negative correlation. **(E)** Scatter plot depicts the correlation of LYVE1+ macrophages with scores and cell expression in the skeletal muscle microenvironment. **(F)** Representative Periodic acid-Schiff (PAS) staining pathological images of tissue samples collected from the groups classified as young, aged, and sarcopenia (scale bar = 100 μm). **(G)** Representative images of immunohistochemical (IHC) staining for CD68 in human skeletal muscle tissue from the young, aged, and sarcopenia groups (scale bar = 100 μm). The black arrow points to the macrophages.

The enrichment of inflammatory signaling pathways is often accompanied by the infiltration of immune cell populations. To gain further insights into the sarcopenia microenvironment, we categorized samples based on the prevalence of macrophage subpopulations, enabling us to investigate muscle immune responses under conditions of both high and low macrophage abundance within the microenvironment. In the subsequent analysis, the samples were divided into two groups based on LYVE1 expression levels: LYVE1-high (above the median) and LYVE1-low (at or below the median). To investigate the immune microenvironment in different states, we calculated the percentages of immune cell types in each sample, and compared the results among the young, aged and sarcopenia groups as well as between the LYVE1-high and LYVE1-low groups (**Figure 6A**). Overall, there were no significant differences observed between the LYVE1-high and LYVE1-low groups in most immune cell types except B cells memory, NK cells resting and M2 macrophages, indicating the activation of NK cells and B cells by high abundance of macrophages. Additionally, there was an increasing trend in monocytes in the LYVE1-high group (**Figure 6B**). To examine the variations in immune microenvironment compositions between the LYVE1-high and LYVE1-low groups, we utilized the ssGSEA algorithm to assess the enrichment score of genes associated with M0, M1 or M2-like macrophages as well as inflammation, and the expression of distinct immune cell types (**Figure 6C**). Our findings revealed that the majority of aged and sarcopenic individuals belonged to the LYVE1-high group, with an increase in inflammation-related gene expression. Additionally, the LYVE1-high group exhibited an increase in the expression of marker genes for M0, M1, and M2-like macrophages. LYVE1 is identified as a marker gene for tissue-resident macrophages at various stages of differentiation, as evidenced by its distinct expression in muscle macrophages (**Supplementary Figure S1**). In the LYVE1-high group, we observed a higher presence of immune cells, FAP cells and endothelial cells in the skeletal muscle microenvironment. The correlations among cells in the LYVE1-high and LYVE1-low groups were found to be different. For instance, we found a negative correlation between M1 macrophages and NK cells in the LYVE1-low group, while a positive correlation was observed in the LYVE1-high group. Additionally, the correlation between type I and type IIa cells was stronger in the LYVE1-high group compared to the LYVE1-low group (**Figure 6D**). Stromal and basal scores showed a positive correlation with the expression of LYVE1+ macrophage, indicating an increase in basal cells and extracellular matrix in the presence of macrophages. Moreover, we investigated the correlation between LYVE1+ macrophages and FBN1+FAP cells, as well as myeloid cells (**Figure 6E**). To validate our findings, we conducted histological staining (**Figures 6F**, **G**). PAS staining was utilized to visualize the distribution of glycogen in muscle tissue. The results clearly indicated that elderly individuals and patients with sarcopenia exhibited lower levels of muscle glycogen compared to young people, which aligned with previous studies (38). CD68 antibodies were employed to label macrophages. IHC analysis revealed a small number of macrophages scattered within the muscle interstitium of young samples. In contrast, a larger number of macrophages were found to be distributed in both aged and sarcopenic samples. These findings strongly suggest a correlation between macrophages and muscle fibrosis, with bone marrow-derived monocytes potentially serving as an important source of LYVE1+ macrophages.

### 3.7 Gene functional annotation analysis of modules associated with macrophages

**Figure 7:**
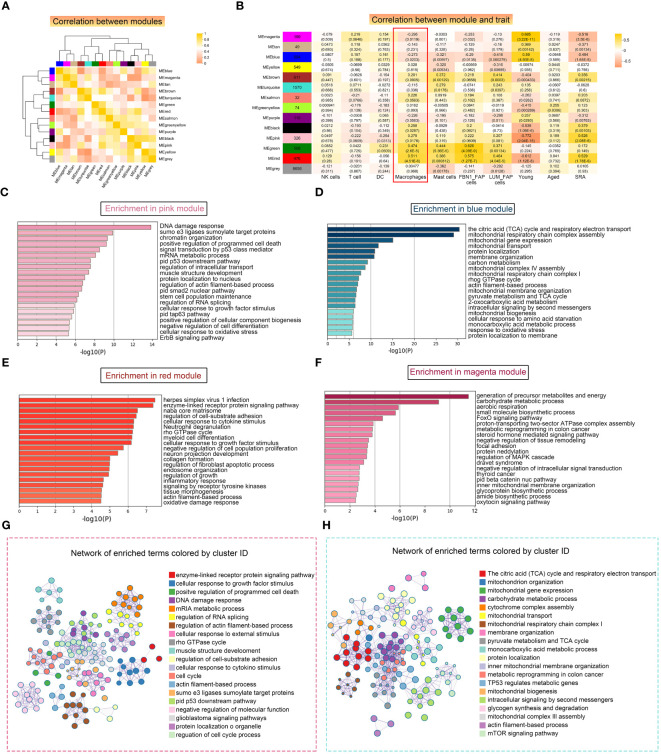
Weighted correlation network analysis (WGCNA) revealed unique functional gene modules associated with LYVE1+ macrophages. **(A)** Heatmap demonstrating the Pearson correlation coefficients of eigengenes of co-expressed gene modules. A total of 14 modules were identified with highly correlated gene expression patterns. **(B)** Correlations between WGCNA modules and microenvironment cell type and age and disease state. Each cell represents Person’s correlation coefficient and p-value. **(C–F)** The results of Metascape for genes in the pink **(C)**, blue **(D)**, red **(E)** and magenta **(F)** modules were represented using a horizontal bar chart. **(G, H)** Enrichment clustering network analysis in the Metascape database of red and pink merged modules **(G)** and blue and magenta merged modules **(H)**.

Next, we conducted WGCNA to identify functional modules that were closely associated with macrophages. A total of 14 modules were detected, with the magenta and blue modules exhibiting the highest negative correlation with macrophages. Additionally, we selected two modules, pink and red, which showed a strong positive correlation with macrophages, for further investigation (**Figures 7A, B**). The correlation analysis revealed that the magenta and blue modules had a negative correlation with sarcopenia, while the pink and red modules showed a positive correlation with sarcopenia, indicating consistency between sarcopenia and macrophages. The pink module consisted of 299 genes primarily involved in cellular regulation, signaling pathways, intracellular mechanisms, and structural regulation (**Figure 7C**). On the other hand, the red module was enriched in pathways associated with the extracellular matrix and fibrosis (**Figure 7E**). Further analysis of genes in these two modules demonstrated significant enrichment in various pathways, including muscle structure development, DNA damage response, and cell cycle. The results were visualized using Cytoscape (**Figure 7G**). Next, we conducted an analysis on the modules that displayed a negative correlation with macrophages. Through Metascape analysis, we found that genes within the blue module were primarily enriched in pathways related to mitochondrial metabolism and function, and genes within the magenta module were found to be involved in various metabolic pathways and signal transduction pathways (**Figures 7D, F**). Upon merging the two modules and conducting enrichment analysis, it was noticed that genes showing a negative correlation with macrophages were notably enriched in pathways associated with mitochondria (**Figure 7H**).

### 3.8 Functional association network analysis of vital immune cells

**Figure 8:**
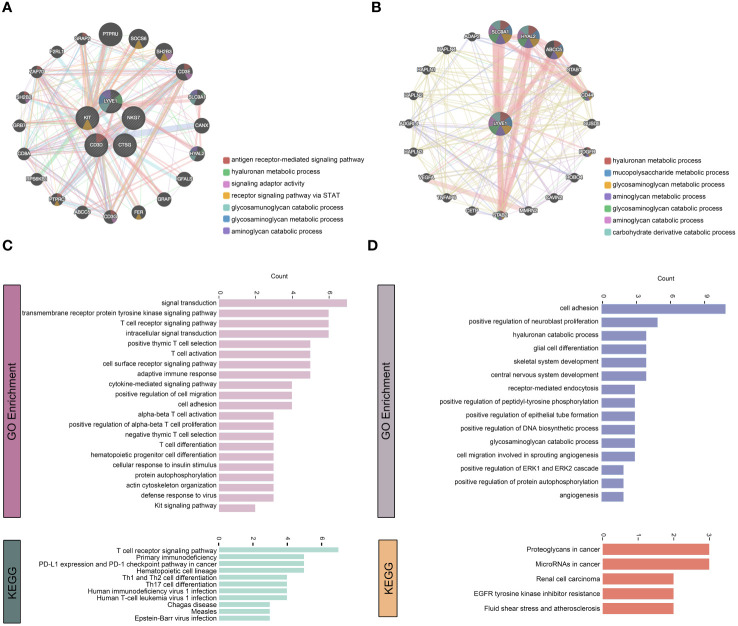
Functional association network analysis of vital immune cells. **(A, B)** Network inference carried out by examining the 5 genes that characterize T cells, NK cells, DCs, mast cells, and macrophages through GeneMANIA **(A)**. Additionally, only the LYVE1 gene considered **(B)**. The edges in the network representing interactions and color-coded based on the specific function of interaction occurring between the genes. Nodes represent genes associated with the hub genes. **(C, D)** GO and KEGG analyses on the associated genes to determine their functions.

We utilized GeneMINIA to predict the protein-protein interaction network of vital immune cells, particularly macrophages, in the skeletal muscle (**Figures 8A, B**). Next, we performed GO and KEGG analyses to interrogate the biological processes and pathways these relative genes involved in (**Figures 8C, D**). The identified immune cell-related pathways encompassed various immune system processes, signal transduction, adaptive immune response, as well as several T-cell differentiation and activation pathways. Additionally, the GO terms and KEGG pathways related to macrophages included pathways associated with cell signaling and tissue development, such as angiogenesis.

## 4 Discussion

Age-related decline in skeletal muscle mass and function is a multifactorial phenomenon, characterized by the loss of muscle fibers and the atrophy of remaining fibers (39, 40). Previous studies have highlighted the role of immune function changes in aging muscle (17, 41, 42), but the specific alterations in the immune microenvironment of skeletal muscle with age have yet to be fully understood. In this study, we utilized snRNA-seq analysis in combination with bulk RNA-seq to investigate the differences in cell composition and immune microenvironment between young and aged skeletal muscle tissue. Since macrophages were found to be the predominant immune cell population, we further identified a specific marker gene LYVE1 for macrophages and clustered them into subgroups. Additionally, we explored changes in cell-cell interactions between aged and young muscle. Furthermore, by analyzing bulk RNA-seq data, we examined the gene expression signature, functional differences, and infiltration characteristics of the young, aged, and sarcopenia groups, as well as the LYVE1-high and LYVE1-low groups. Our findings offered valuable insights into potential therapeutic strategies for sarcopenia, including targeting specific immune cell populations or modulating their activity. Notably, our results suggested a close association between macrophages and metabolic and functional alterations, as well as injury repair, in aging muscle.

Our analysis revealed that the sarcopenia group exhibited upregulation of several immune-related pathways, such as JAK-STAT, ERBB, and IL-2/4 signaling pathways, in comparison to young controls. Previous studies have also linked these pro-inflammatory pathways to age-related muscle atrophy (43, 44). We also discovered a crosstalk between immune cells and various non-immune cells within the muscle microenvironment. The results indicated that immune cells received signals from fibroblasts, endothelial cells and other cell types, underscoring the significance of immune cells in the skeletal muscle microenvironment. Macrophages were found to be the predominant immune cell type in damaged muscles and played a crucial role in both the inflammation process and its resolution, contributing significantly to muscle repair (45, 46). In response to anti-inflammatory cytokines, M1 macrophages transform into M2 macrophages in the later stages of skeletal muscle injury repair. Subsequently, these M2 macrophages along with resident M2 macrophages, facilitate the repair and regeneration processes by enhancing myoblast differentiation and vascularization, and stimulating FAP to generate extracellular matrix. But macrophages in aged muscle are inclined to be polarized to a proinflammatory-phenotype, thereby negatively impacting the repair and regeneration capabilities of damaged muscle (47). Zhang C et al. identified and characterized a unique macrophage subpopulation that specifically expressed IFN-responsive genes in mice, which could promote the proliferation and differentiation of satellite cells (48). A recent study has further shown that aging leads to a decrease in the expression of MANF in macrophages, which in turn affects the regeneration ability of muscles (49). In our study, snRNA-seq analysis revealed that the skeletal muscle immune microenvironment consisted of a diverse range of innate and adaptive immune cells, with macrophages comprising the largest proportion. Additionally, other immune cells were also involved in chronic muscle inflammation. Our research has revealed a significant correlation between DCs and macrophages. Similar to macrophages, DCs also function as potent antigen-presenting cells. When confronted with injury, DCs are activated to induce adaptive immunity, while macrophages initiate an inflammatory response (50). Abnormal accumulation of DC has been reported in inflammatory myopathic muscles (51). Within the muscle microenvironment, various immune cells exhibit distinct metabolic pathways and biological functions. M1 macrophages, for instance, are associated with the synthesis of collagen fibers and play a role in the TGF-beta signaling pathway. DCs are primarily involved in cell killing and sphingolipid metabolism signaling pathways. NK cells are predominantly enriched in the cytolysis pathway, while T cells are closely associated with skeletal muscle differentiation. Treg cells have been reported to enhance repair in chronically injured muscle because the depletion of Treg promotes the macrophages to switch from a pro-inflammatory to an anti-inflammatory phenotype (52). Rebalancing the interactions between immune cells and other tissue cells, as well as among immune cells, may offer potential benefits for patients with sarcopenia.

As the most abundant type of immune cell in skeletal muscle, macrophages play a crucial role in maintaining muscle homeostasis. Resident macrophages in various tissues have unique chromatin landscapes and transcriptional profiles, which depend on their microenvironment and functional condition (53). For this reason, there is currently no standardized macrophage signature. In the past, CD68 and F4/80 were established markers for resting M0 macrophages. Macrophage activation is triggered by extracellular and intracellular stimuli, leading to two main phenotypes: inflammatory (M1 and M2b) and wound resolution (M2a, M2c, and M2d) (54). These phenotypes exhibit distinct functional and metabolic characteristics. M1 macrophages are characterized by high expression levels of CD40 and CD86 and low expression levels of CD163, while functional phenotype of M2 macrophages are characterized by low expression levels of CD86 and high expression levels of CD163. Since macrophages in different tissues have different functions and secrete different factors, this study identified LYVE1 as a marker for specific expression in macrophages within the skeletal muscle microenvironment. Similar findings were also reported by Krasniewski LK et al. in mouse muscles (55). In this study, we performed bulk RNA-seq analysis and utilized LYVE1 as a macrophage marker. We divided the samples into high and low expression groups for LYVE1, and observed that the LYVE1+ macrophage enrichment group exhibited elevated expression levels of the classic M0, M1 and M2 subpopulation. Further transcriptomic analysis revealed that the populations with high expression levels of LYVE1+ macrophages displayed increased expression of genes related to fibrosis and muscle development, indicating the significant role of macrophages in the repair process of injured muscle.

Our study has also identified four distinct macrophage subpopulations based on marker gene expression, which may potentially serve different functional roles in the muscle immune environment and have diverse origins. The LYVE1^lo^MHC-II^h^i subpopulation exhibits enrichment in pathways associated with innate immune responses and antigen presentation, indicating a stronger pro-inflammatory phenotype. On the other hand, the LYVE1^hi^MHC-II^lo^ subpopulation demonstrates upregulation of metabolic pathways, suggesting its involvement in tissue remodeling. The LYVE1^lo^MHC-II^lo^TSPO+ subpopulation, characterized by an enrichment of extracellular matrix tissue genes, has been identified as closely linked to muscle fibrosis and may contribute to the pathological accumulation of fibrosis seen in sarcopenia. Among these subpopulations, we noticed an increase in the expression of LYVE1^hi^MHC-II^lo^ subpopulation in aged muscle. In a study conducted on mice, it was discovered that the LYVE1^lo^MHC-II^hi^ cells originated entirely from hematopoietic stem cells (HSCs), while the LYVE1^hi^MHC-II^lo^ subpopulation consisted of an equal proportion of cells derived from HSCs and non-HSCs (56), which may suggest their different origins.

Currently, targeting immune cell-related pathways within the microenvironment has emerged as a crucial therapeutic strategy for sarcopenia. For instance, supplementing MANF protein can promote the transformation of macrophages from a pro-inflammatory phenotype to a repair phenotype, thus facilitating aging muscle regeneration and regulating inflammation and tissue homeostasis (49). IL-15 has also been identified as a promising therapeutic target for mitigating inflammation-induced skeletal muscle atrophy (57). Further understanding the distinct functions and origins of macrophage subpopulations could offer valuable insights for developing treatments that target specific pro-inflammatory or fibrotic phenotypes.

This study has a few limitations. Firstly, the sample size could be increased to provide more robust results. In particular, the sample size for snRNA-seq needs to be expanded. Additionally, further experiments are needed to verify the efficiency of LYVE1 as a marker gene for skeletal muscle macrophages. While macrophages in the skeletal muscle microenvironment have been categorized, the specific subpopulations that are closely associated with the development of sarcopenia and their corresponding targets still require further investigation. Moreover, human muscle transcriptome exhibits sex-specific changes with aging (58, 59). In addition, a correlation between the incidence of sarcopenia and age is observed as previously reported (5, 60). In this study we were unable to exclude the effect of gender on our results, and our study lacked an age-matched group to compare sarcopenia in younger adults versus young individuals, which will be addressed in our subsequent research.

## 5 Conclusion

In this study, we have identified LYVE1 as a marker gene for tissue-resident macrophages in the skeletal muscle microenvironment across different ages and disease states. Our findings suggest a correlation between LYVE1+ macrophage expression and sarcopenia, as well as significant functional and metabolic differences between the high and low LYVE1 expression groups. Furthermore, our snRNA-seq analysis has uncovered new clustering patterns in human skeletal muscle macrophages. Additionally, we have observed variations in the skeletal muscle microenvironment among aged and young individuals and those with sarcopenia. Overall, our research provides valuable insights into potential strategies for treating sarcopenia by targeting macrophages.

## Data availability statement

The data that support the findings of this study are available in the GEO dataset at https://www.ncbi.nlm.nih.gov/geo/, accession number GSE167186. Further inquiries can be directed to the corresponding authors.

## Ethics statement

The studies involving humans were approved by the Ethics Committee of Ruijin Hospital, Shanghai Jiao Tong University School of Medicine. The studies were conducted in accordance with the local legislation and institutional requirements. The participants provided their written informed consent to participate in this study.

## Author contributions

LS: Investigation, Writing – original draft. YZ: Methodology, Validation, Writing – original draft. JZ: Data curation, Investigation, Methodology, Writing – original draft. YY: Investigation, Software, Writing – original draft. LL: Methodology, Writing – original draft. NL: Formal analysis, Writing – review & editing. YG: Validation, Writing – review & editing. XX: Data curation, Methodology, Writing – original draft. QB: Data curation, Writing – review & editing. LJ: Writing – review & editing. WH: Writing – review & editing, Funding acquisition.
