# Supplementary material for: Characterizing the skeletal muscle immune microenvironment for sarcopenia: insights from transcriptome analysis and histological validation
Source: Front Immunol. 2024 Jul 4;15:1414387. doi: 10.3389/fimmu.2024.1414387 (PMC11254692; doi:10.3389/fimmu.2024.1414387)
Supplement: Supplementary file 1 [file DataSheet_1.docx]

**SUPPLEMENTARY TABLE 1**

**Primary antibodies detailed information**

| antigen | dilution/concentration | Cat. # | | Company and Nation | |
| --- | --- | --- | --- | --- | --- |
| CD45 | 1:100 | 60287-1-Ig | Proteintech, China | |  |
| CD3 | undiluted | GA503 | Dako, Denmark | |  |
| CD68 | undiluted | GA613 | Dako, Denmark | |  |
| CD56 | 1:100 | IR628 | Dako, Denmark | |  |
| CD117 | undiluted | KIT-0029 | MXB Biotechnologies, China | |  |
| α-SMA | 1:500 | GB12044 | Servicebio, China | |  |

**SUPPLEMENTARY FIGURE 1**

**Dot plots representing the expression levels of LYVE1 and PTPRC for each cell cluster (A) and immune cell cluster (B).**


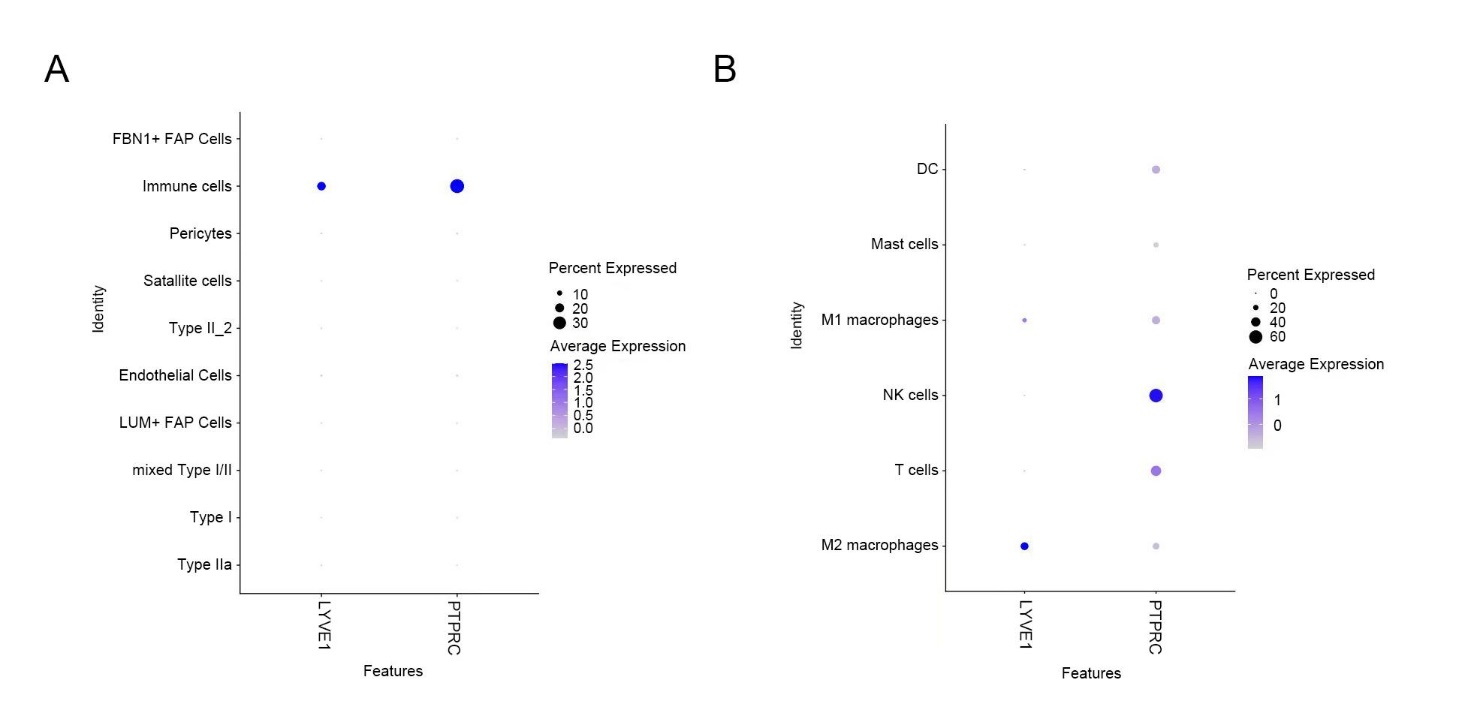


**SUPPLEMENTARY FIGURE 2**

**(A-C) Quantification of IHC staining positive area for Figure 2I (A), J (B), and K (C). (D) quantification of IF staining positive area for Figure 4G. (E)** **The LYVE1hiMHC-IIlo subpopulation proportion in the young and aged group.**


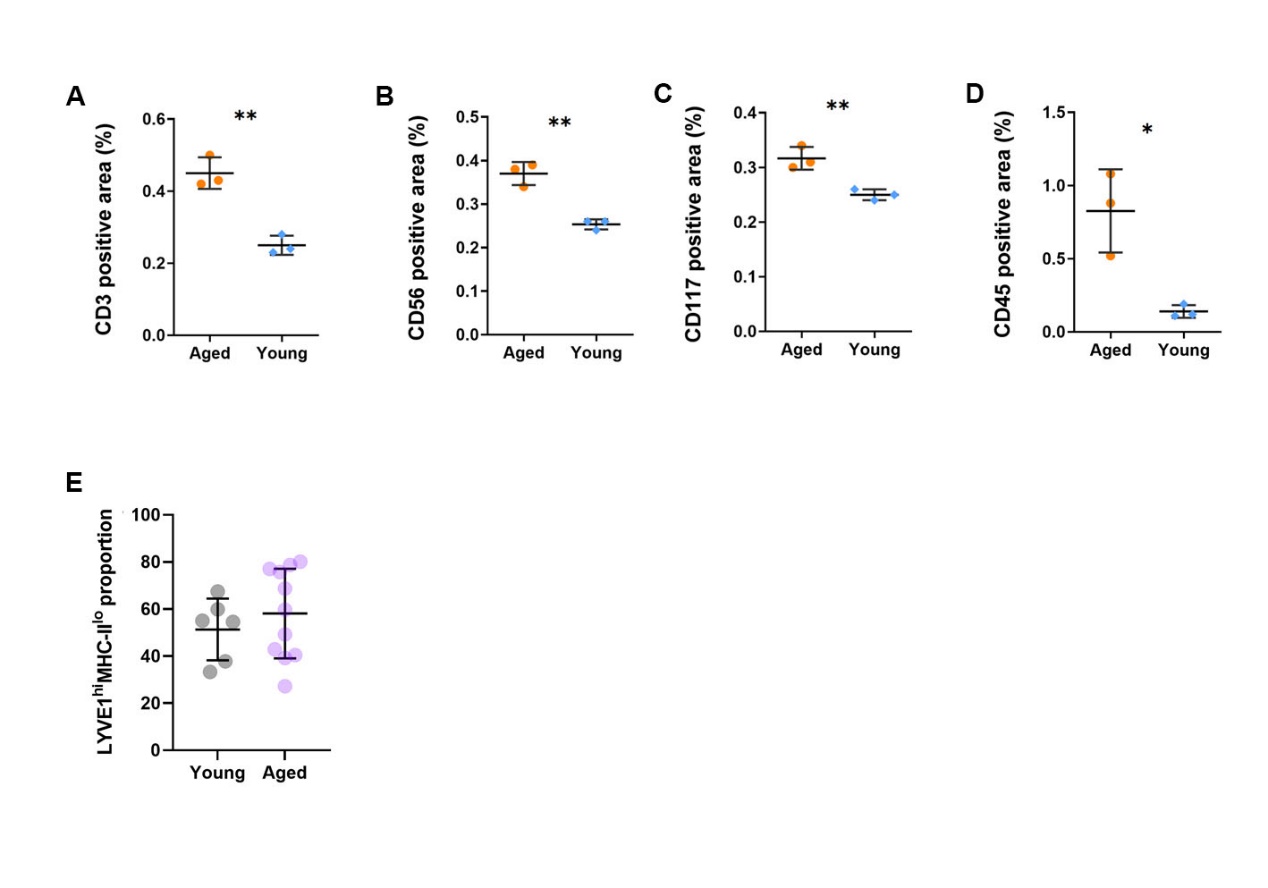


**SUPPLEMENTARY FIGURE 3**

**(A) Grid plot representing the expression of selected marker genes for each cluster in the young group and aged group. (B) Differentially expressed genes (DEGs) in each cell type in aged versus young skeletal muscle.** **(C) Grid plot depicting the expression of the top 10 upregulated genes identified in each cell cluster.**

**
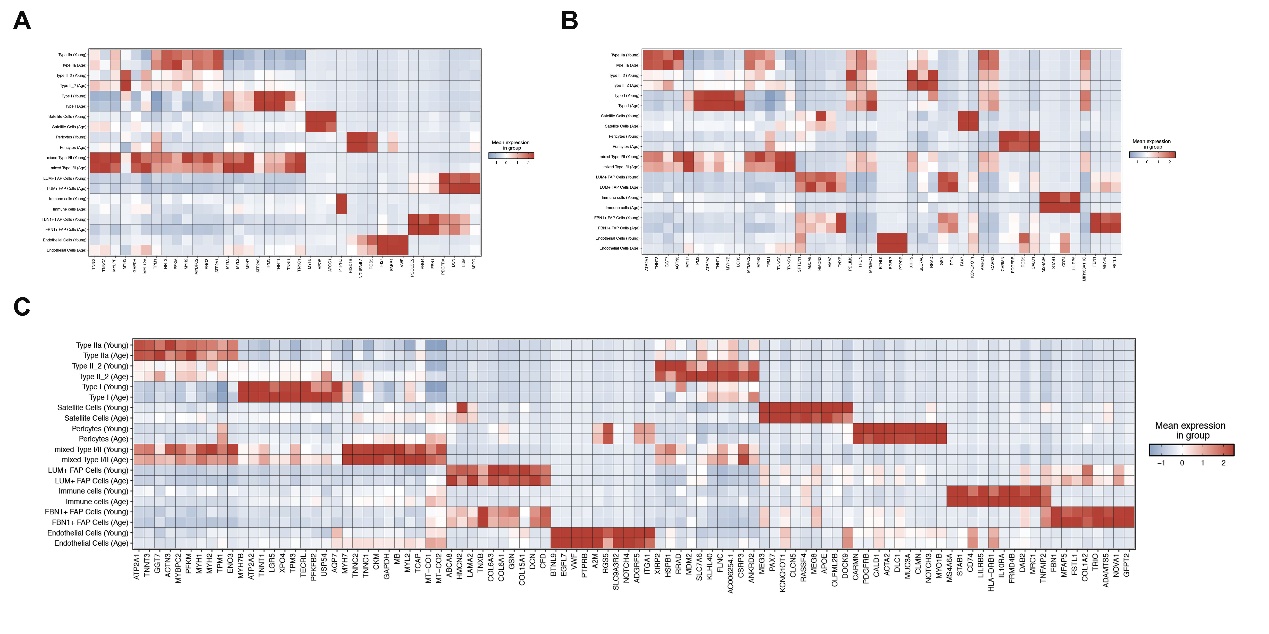
**
